# A Novel Optimal Layout Method for Rain Gauge Network Based on Mutual Information Entropy and Deep Learning Model

**DOI:** 10.3390/s26113532

**Published:** 2026-06-03

**Authors:** Yanyan Huang, Xin Lu, Han Luo, Bin Liu, Rui Wang

**Affiliations:** 1 School of Software Engineering, Chengdu University of Information Technology, Chengdu 610225, China; 2 Sichuan Research Institute of Water Conservancy, Chengdu 610072, China

**Keywords:** rain gauge network optimization, remote sensing precipitation data, CNN-LSTM model, mutual information entropy, hybrid optimization algorithm, upper reaches of the Tuojiang River

## Abstract

Rain gauge networks are the core infrastructure for hydrological and water resource monitoring, flood control and disaster mitigation early warning, and water resource planning and regulation. The rationality of their layout directly determines the accuracy, representativeness, and economy of regional precipitation data acquisition. Considering that information entropy can accurately characterize the spatial distribution law and information complexity of rainfall, and spatiotemporal deep learning models have strong capabilities in fitting spatiotemporal features, this paper couples mutual information entropy with a spatiotemporal deep learning model and proposes a novel optimal layout method for rain gauge networks. Daily observed rainfall data from 50 ground-based rain gauges in the upper reaches of the Tuojiang River during 2015–2024, as well as the PERSIANN-CCS remote sensing precipitation product for the same period, were used in the study. A CNN-LSTM spatiotemporal deep learning model integrating spatial features and temporal dependence was constructed, coupled with the mutual information entropy index, and the GA-PSO hybrid optimization algorithm was applied for solution. The superiority of the proposed method was verified by comparison with the calculation results of the traditional mutual information entropy-based greedy optimization algorithm. The results show that the hybrid optimization algorithm driven by the spatiotemporal deep learning model coupled with mutual information entropy is significantly superior to the comparison algorithm in terms of the rationality of the station network structure, the ability to characterize spatial rainfall distribution, the control of average relative error, and the improvement of total information entropy. After optimization, the number of rain gauges in the upper reaches of the Tuojiang River can be reduced from 50 to 25. While greatly reducing the number of stations, the optimized network can still relatively accurately reflect the spatiotemporal characteristics of rainfall in the basin, which can provide a theoretical basis and technical support for the optimal layout of basin rain gauge networks and water resource management.

## 1. Introduction

Precipitation is the core element of the basin hydrological cycle, and accurate rainfall monitoring is an important foundation for flood forecasting, water resource regulation, water conservancy project operation, and aquatic ecological protection [1]. The latest report from the World Meteorological Organization (WMO) shows that more than 60% of countries lack a complete ground-based rain gauge network, which seriously restricts the accuracy of global satellite precipitation inversion [2]. As the core carrier of ground rainfall monitoring, the rationality of the rain gauge network layout directly determines the accuracy, completeness, and usability of rainfall data, and has important practical significance for basin hydrological simulation and disaster prevention and control [3]. With the rapid development of deep learning technology, its strong capabilities in feature mining and complex relationship modeling have provided a new technical path for rain gauge network optimization. Compared with traditional methods, deep learning can effectively capture the complex correlation characteristics of spatiotemporal heterogeneity of rainfall [4], and break the assumption limitations of traditional methods on data distribution, significantly improve the scientificity and accuracy of station network optimization, and provide strong support for solving the problem of optimal layout of station networks in complex basins.

Traditional rain gauge network optimization methods are mostly based on information entropy theory, geospatial interpolation [5], expert knowledge method [6], etc., For example, Li et al. [7] proposed a method of maximum information and minimum redundancy for rain gauge network design, taking the maximum internal correlation entropy, minimum redundancy of the network, and maximum mutual information with other stations as the optimization objectives, and applied a greedy algorithm to solve the optimal rain gauge network. Alfonso et al. [8] used the simulated data and measured data of hydrological models to design water level and runoff monitoring station networks with the maximum internal correlation entropy and minimum mutual information entropy of the station network as the optimization objectives, respectively. Huang et al. [9] combined Kriging interpolation and entropy theory, and took the minimum Kriging variance, maximum comprehensive information content of the station network, and maximum spatiotemporal information content as the optimization objectives, and obtained the optimal layout of the rain gauge network in the upper reaches of the Chaobai River. Wallace Chin and Suhaili [10] combined Kriging error uncertainty with MAE, considering local prediction uncertainty and global prediction accuracy, which can conduct a more comprehensive evaluation of the rain gauge network. Haggag et al. [11] used ordinary Kriging and sequential algorithm to evaluate the existing rain gauge network in the arid area of northern Oman, diagnosed its monitoring blind areas and redundant stations, and proposed an optimization scheme of “streamlining redundant stations + redeploying to blind areas”. Skok [12] used analytical expressions to find the relationship between the total number of rain gauges, regional area, and average distance between stations to estimate the layout density of rain gauges. Wang et al. [6] based on GIS technology and spatial optimization methods, evaluated the coverage area of ground rain gauge stations through factors such as topography, and applied it to the layout of the rain gauge network in the Jinsha River Basin. The main core of the algorithms is to calculate indicators such as station information redundancy and spatial uniformity, and use greedy algorithms, genetic algorithms, etc. for station selection and layout adjustment. However, such methods generally have limitations: they need to more fully consider the spatiotemporal dynamic correlation characteristics of rainfall, are prone to fall into local optimal solutions, and have limited optimization effects.

With the development and application of deep learning technology, new methods have been provided for the mining of spatiotemporal characteristics of rainfall. Its core advantage lies in its ability to accurately capture the spatiotemporal heterogeneity and complex correlation characteristics of rainfall, making up for the shortcomings of traditional methods. Shi et al. [13] proposed the ConvLSTM model, which combines convolution with LSTM to effectively capture the spatiotemporal dependence of precipitation, laying a foundation for the mining of spatiotemporal characteristics of rainfall. Shi et al. [14] improved the ConvLSTM structure, further improving the accuracy of short-term precipitation forecasting and providing technical support for rainfall feature prediction in station network optimization. Since then, the application of deep learning models in related fields has been continuously deepened. Chen et al. [15] proposed an innovative “dual-stream” ConvLSTM architecture and 3D-SA-LSTM memory unit to enhance the ability to capture spatiotemporal heterogeneity of rainfall. Zhang et al. [16] clearly elaborated the division of labor between CNN and LSTM: CNN analyzes spatial features, LSTM captures temporal dynamics, and their combination effectively models the local spatiotemporal features of precipitation. Xu et al. [17] pointed out that the CNN-LSTM hybrid structure can accurately track the movement trajectory and intensity changes in precipitation, which are the core spatiotemporal features that need to be captured in rain gauge network optimization, indirectly providing a reference for the optimal layout of station networks.

Remote sensing precipitation products can provide large-scale and continuous grid rainfall data, which effectively makes up for the defects of insufficient spatial coverage and many monitoring blind areas of ground measured data [18]. They have been widely used in the optimization of rain gauge network layout to assist in station site selection and information redundancy evaluation. Yeh et al. [19] applied high temporal resolution radar precipitation data to the information entropy method with minimum internal information redundancy of the station network, and obtained the optimal rain gauge network layout in the study area. Dai et al. [20] applied radar precipitation data and proposed a method for rain gauge layout in the study area based on principal component analysis and clustering method. Principal Components Analysis (PCA) was used to analyze the redundancy of radar grid data and determine the number of rain gauges, and Cluster Analysis (CA) was used to determine potential locations. Hsu et al. [21] developed the PERSIANN algorithm to realize precipitation inversion from satellite infrared data. As a major improved version launched in 2004, PERSIANN-CCS improved the spatial resolution from 0.25° to 0.04° by introducing the “Cloud Classification System”, laying a foundation for the development of remote sensing precipitation products. Zeweldi et al. [22] evaluated the results of PERSIANN-CCS using radar precipitation observation data, and the results showed that the accuracy of PERSIANN-CCS can well capture the pattern of international annual precipitation changes. Cánovas-García et al. [23] evaluated three heavy precipitation events, and the results showed that PERSIANN-CCS well captured the temporal distribution of rainfall.

In view of the shortcomings of existing rain gauge network optimization methods and the advantages of remote sensing precipitation products, this study proposes a rain gauge network optimization framework that couples mutual information entropy, a CNN-LSTM spatiotemporal deep learning model, and a GA-PSO hybrid optimization algorithm based on both remote sensing precipitation products and in situ rain gauge observations. Compared with previous methods that mainly rely on information entropy, geostatistical interpolation, expert knowledge, or greedy search strategies, the proposed method differs in the following aspects. First, multi-source precipitation information is integrated by combining ground-based rainfall observations with PERSIANN-CCS remote sensing precipitation products, thereby improving the spatial coverage of rainfall information used for network optimization. Second, a CNN-LSTM spatiotemporal feature extraction model is introduced to jointly characterize local spatial dependencies and temporal rainfall evolution, overcoming the limitation of traditional entropy-based methods that insufficiently consider dynamic spatiotemporal rainfall correlations. Third, mutual information entropy is coupled with the spatiotemporal features extracted by the deep learning model to construct a comprehensive station evaluation index, which balances information redundancy, monitoring accuracy, and spatial representativeness. Fourth, a GA-PSO hybrid optimization algorithm is adopted to combine the global search capability of genetic algorithms with the local refinement ability of particle swarm optimization, reducing the risk of falling into local optima compared with conventional greedy optimization. Through comparison with a typical mutual information entropy-based greedy algorithm, the proposed framework provides a streamlined and efficient layout scheme for rain gauge networks and offers a methodological reference for basin-scale hydrological monitoring network optimization.

## 2. Materials and Methods

### 2.1. Study Area Overview

**Figure 1:**
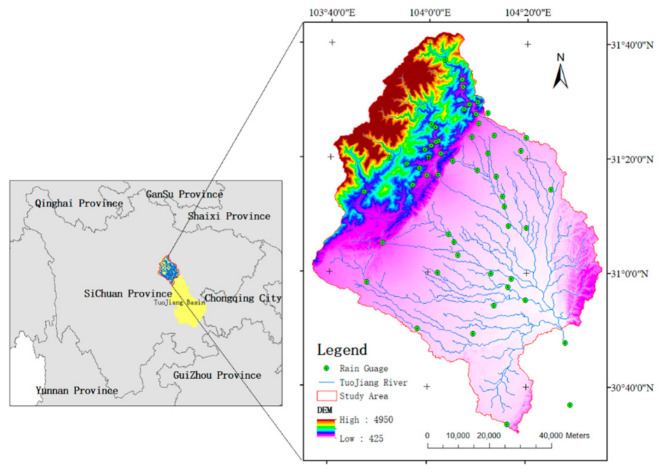
Study area of the upper Tuojiang River Basin.

The upper reaches of the Tuojiang River are selected as the study area. As the upstream section of the Tuojiang River Basin, it serves as the source region of the Tuojiang River, an important tributary of the upper Yangtze River, and constitutes a key part of the ecological security barrier in Southwest China. Furthermore, it is a critical node for flood control, disaster mitigation, and water resource allocation in the Yangtze River Basin. It is of great strategic significance for safeguarding the aquatic ecological balance of the Yangtze River Basin and supporting the sustainable economic and social development of Southwest China. The region is located in the northwestern Sichuan Basin, with a geographical coordinate range of 103°36′–104°35′ E longitude and 30°32′–31°42′ N latitude. The main stream length is approximately 127 km, and the drainage area covers 6378.02 km^2^. The terrain is dominated by mountains and hills, with a high northwest and low southeast topographic pattern and a large elevation difference ranging from 500 m to 3500 m. The climate features significant vertical differentiation, and the spatiotemporal distribution of rainfall is highly uneven. The annual precipitation is about 800–1500 mm, of which more than 70% occurs in the flood season (June–September). Rainstorms occur frequently, making the area a national key region for flood control and disaster reduction. At present, 50 ground rain gauges are distributed in the basin with uneven spatial arrangement: some areas are densely covered by stations, while monitoring blind spots exist in other regions. The spatial location and topographic setting of the study area are shown in Figure 1.

### 2.2. Data Sources and Preprocessing

The precipitation data used in the study include two types: daily rainfall data from ground observation stations and PERSIANN-CCS remote sensing precipitation products during 2015—2024, which together form a multi-source rainfall dataset covering the upper reaches of the Tuojiang River Basin. Among them, the measured rainfall data are derived from 50 ground-based rain gauges in the upper reaches of the Tuojiang River, with a daily time scale. The data were subjected to quality control: abnormal values were eliminated using the 3σ principle, and missing values were interpolated by combining the adjacent station interpolation method [24] and the time series trend method [25]. After interpolation, the data completeness reached more than 98%, which can meet the accuracy requirements for model training and station network optimization. The remote sensing precipitation product is PERSIANN-CCS, with a spatial resolution of 0.04° × 0.04° and an hourly time resolution, which can provide near-real-time satellite precipitation observations [26]. It can capture the details of spatiotemporal evolution of precipitation more precisely and has been widely applied in flood early warning and hydrological model simulation studies. (The data were downloaded from the official data platform: https://persiann.eng.uci.edu/CHRSdata/PERSIANN-CCS/daily/, accessed on 15 February 2024). After preprocessing (including spatial clipping of the study area, masking processing, and grid data extraction), the daily precipitation products of the study area from 2015 to 2024 were obtained. With the help of ArcGIS 10.8.1 software, the remote sensing data were projected to the same coordinate system (WGS84_UTM_Zone_48N) as the measured data to construct a grid rainfall dataset matching the measured stations. Meanwhile, the measured data were used to verify the accuracy of the remote sensing data. The results show that the correlation coefficient (R) between the remote sensing data and the measured data is 0.82, and the root mean square error (RMSE) is 2.31 mm/d, which meets the data source requirements for station network optimization.

### 2.3. Research Methods

**Figure 2:**
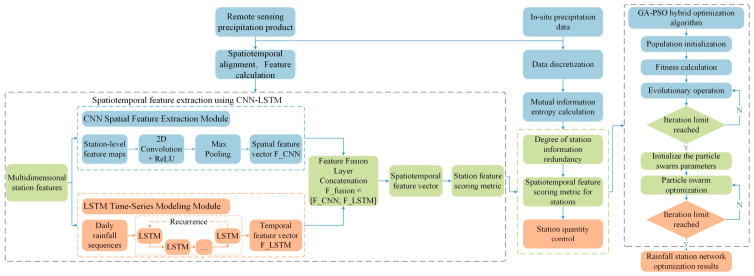
Flowchart of the proposed rain gauge network optimization method.

The rain gauge network optimization method proposed in this study includes four core links: basic feature calculation, CNN-LSTM spatiotemporal deep learning model construction, mutual information entropy calculation, comprehensive index calculation, and hybrid optimization algorithm solution. The technical route is shown in Figure 2.

(1) Calculation of Basic Characteristic Values

Firstly, data alignment and sorting are carried out. The temporal scales of observed rainfall data and remote sensing data are unified, and the remote sensing grid data are interpolated to the same spatial locations as the ground stations via spatial interpolation, so as to construct a paired “observed-remote sensing” dataset and eliminate the influence caused by spatial scale differences.

Secondly, feature vector calculation for each station is performed. The rainfall time-series features (e.g., mean, variance, extreme value, coefficient of variation, etc.), spatial location features (longitude, latitude, elevation, slope, aspect, topographic conditions, etc.) of each station, as well as grid features corresponding to remote sensing data (grid rainfall mean, grid rainfall coefficient of variation, information entropy, etc.) are calculated to form a multi-dimensional input feature vector, preparing data for model training.

Finally, dataset partitioning was implemented. The dataset was divided into training, validation, and test sets at a ratio of 7:2:1, which were used for model training, parameter tuning, and performance evaluation, respectively. It should be noted that hydrological station network data cannot be randomly partitioned; instead, the principle of spatiotemporal consistency should be followed, i.e., maintaining the continuity of rainfall time series and the correlation of spatial distribution. Therefore, the data were divided into continuous temporal segments to preserve the seasonal pattern of rainfall and the continuity of rainstorm processes.

To further reduce the risk of data leakage between adjacent temporal partitions, a purging strategy was applied around each partition boundary. A temporal buffer with a length equal to the model’s look-back window was reserved before and after each splitting point. Any samples whose look-back windows or prediction targets overlapped across partitions were excluded, ensuring that rainfall events spanning a partition boundary were not simultaneously used for model fitting and independent evaluation. Meanwhile, all input features were standardized to the interval [0, 1] to avoid interference from inconsistent feature dimensions during model training.

To further evaluate the cross-year robustness of the proposed optimization framework and to avoid potential overfitting to specific climatic cycles within the 10-year dataset, a leave-one-year-out validation strategy was further introduced. Specifically, one year from 2015 to 2024 was sequentially excluded from model training and station-network optimization and was used only for independent testing, while the remaining nine years were used for CNN-LSTM model training, station evaluation indicator calculation, and station-network optimization. This process was repeated ten times until each year had been used once as the independent test year. The mean relative error (MRE) between the reconstructed precipitation from the optimized station network and that from the original station network was adopted to evaluate the cross-year generalization performance. The traditional mutual-information-entropy-based greedy optimization method was also tested under the same leave-one-year-out setting to provide a consistent comparison.

(2) Construction of the CNN-LSTM Spatiotemporal Deep Learning Model

The CNN-LSTM model integrates the spatial feature extraction capability of Convolutional Neural Network (CNN) and the time-series modeling capability of Long Short-Term Memory network (LSTM), which can accurately capture the spatiotemporal correlation characteristics of rainfall and provide a reliable basis for the comprehensive evaluation of stations. The main aim of using CNN-LSTM in this study is to extract representative spatiotemporal rainfall features for station evaluation and network optimization, rather than merely to build a standalone rainfall prediction model. The overall model adopts a structure consisting of an Input Layer, a CNN Spatial Feature Extraction Module, an LSTM Time-Series Modeling Module, and a Feature Fusion Layer. These modules work collaboratively and progressively to mine rainfall spatiotemporal features and quantitatively evaluate station representativeness. Although Transformer-based models have shown strong potential in sequence modeling and spatiotemporal prediction tasks, the CNN-LSTM architecture was selected in this study considering the data scale, interpretability, and integration with the subsequent optimization framework. The available dataset consists of daily observations from 50 rain gauges over a 10-year period, which is relatively limited for training data-intensive Transformer-based models. In contrast, CNN-LSTM provides a clear division of functions: the CNN module extracts local spatial dependencies among stations, whereas the LSTM module captures temporal rainfall evolution. This structure is consistent with the spatiotemporal characteristics of rainfall monitoring and facilitates coupling with mutual information entropy and the GA-PSO hybrid optimization algorithm. The model structure and logic are described as follows:

1. Input layer. The multidimensional features constructed during the basic feature calculation step, including station spatial features, remote sensing grid features, and rainfall time-series features, are standardized and then fed into the CNN and LSTM modules.
2. CNN Spatial Feature Extraction Module. In this study, a two-dimensional convolutional neural network (2D-CNN) is employed to extract spatial features from station-level feature maps. Unlike a 3D-CNN, which is typically used for volumetric or spatiotemporal tensor data, the adopted 2D-CNN operates on two-dimensional feature maps constructed from station-related spatial attributes and remote sensing precipitation descriptors, including longitude, latitude, elevation, slope, aspect, and grid-based precipitation features. The 2D-CNN module captures local spatial dependencies, spatial gradients, and rainfall clustering patterns among neighboring stations through two-dimensional convolution (Equation (1)) and pooling operations (Equation (2)). The extracted spatial representations are subsequently fused with the temporal features learned by the LSTM module.

(1) $$Conv\left(X\right)=\sigma \left(\sum_{k=1}^{K}W_{k}*X+b_{k}\right)$$

(2) $$MaxPool\left(X\right)={max}_{i\in pool}X(i,j)$$

where X denotes the two-dimensional input feature map of the current layer, $W_{k}$ is the k-th convolution kernel, ∗ represents the 2D convolution operation, $b_{k}$ is the bias term, σ is the ReLU activation function defined as $\sigma \left(x\right)=max(0,x)$, K is the number of convolution kernels, and Pool denotes the pooling window.

3. LSTM Time-Series Modeling Module (Equation (3)). Focusing on the temporal dependence of rainfall, this module captures the long-term evolutionary patterns of rainfall (including seasonal cycles, continuity of rainstorm processes, interannual variation trends, etc.) through gating mechanisms, avoiding the vanishing gradient problem of traditional Recurrent Neural Networks (RNNs).

$$Forget\;gate:f_{t}=\sigma (W_{f}\cdot \left[h_{t-1},x_{t}\right]+b_{f})$$

$$Input\;gate:i_{t}=\sigma (W_{i}\cdot \left[h_{t-1},x_{t}\right]+b_{i})$$

$$Cell\;state\;update:{\tilde{C}}_{t}=tanh(W_{C}\cdot \left[h_{t-1},x_{t}\right]+b_{C})$$

$$Candidate\;cell\;state:C_{t}=f_{t}⊙C_{t-1}+i_{t}⊙{\tilde{C}}_{t}$$

$$Output\;gate:o_{t}=\sigma (W_{o}\cdot \left[h_{t-1},x_{t}\right]+b_{o})$$

(3) $$Hidden\;state\;output:h_{t}=o_{t}⊙tanh(C_{t})$$

where $h_{t-1}$ is the hidden state output at the previous time step, $x_{t}$ is the input at the current time step, $W_{f}$, $W_{i}$, $W_{C}$, $W_{o}$ are weight matrices, $b_{f}$, $b_{i}$, $b_{C}$, $b_{o}$ are bias terms, ⊙ denotes element-wise multiplication, σ is the Sigmoid activation function, and tanh is the hyperbolic tangent activation function.

4. Feature Fusion Layer. The spatial feature vector output by the CNN module and the temporal feature vector output by the LSTM module are concatenated and fused (Equation (4)) to construct a unified spatiotemporal fusion feature vector, which comprehensively represents the spatiotemporal representativeness of each station.

(4) $$F_{fusion}=[F_{CNN};F_{LSTM}]$$

where $F_{CNN}$ is the spatial feature vector output by the CNN module, and $F_{LSTM}$ is the temporal feature vector output by the LSTM module. Feature concatenation realizes deep fusion of rainfall spatiotemporal features and reflects the spatiotemporal representativeness of stations.

5. Model Training and Hyperparameter Optimization. The Adam optimizer is adopted with an initial learning rate of 0.001. This optimizer features fast convergence and strong stability, and can effectively alleviate local optima during model training. The loss function uses Mean Squared Error (MSE), which takes the error between estimated rainfall and observed rainfall at stations as the optimization objective, expressed as follows:

(5) $$MSE=\frac{1}{n}\sum_{1}^{n}{\left(y_{i}-\hat{y_{i}}\right)}^{2}$$

where $y_{i}$ is the measured rainfall characteristic value at the station, and $\hat{y_{i}}$ is the predicted value of the model. The batch size is set to 32 to balance training efficiency and stability, avoiding insufficient memory caused by an overlarge batch or training fluctuation caused by an undersize batch. The maximum training epoch was increased to 300, with an early stopping strategy adopted simultaneously. Training was terminated if the validation loss did not improve for 30 consecutive epochs, thereby preventing unnecessary training and reducing the risk of overfitting. The model corresponding to the minimum validation loss was selected as the final model to balance fitting capacity and generalization performance. The parameter update formula of the Adam optimizer is:

$$m_{t}=\beta_{1}m_{t-1}+\left(1-\beta_{1}\right)\nabla L$$

$$v_{t}=\beta_{2}v_{t-1}+\left(1-\beta_{2}\right){\left(\nabla L\right)}^{2}$$

$${\hat{m}}_{t}=\frac{m_{t}}{1-\beta_{1}^{t}}\;\;\;{\hat{v}}_{t}=\frac{v_{t}}{1-\beta_{2}^{t}}$$

(6) $$\theta_{t}=\theta_{t-1}-\eta \cdot \frac{{\hat{m}}_{t}}{\sqrt{{\hat{v}}_{t}}+\epsilon }$$

where $m_{t}$ and $v_{t}$ are the first-order and second-order moment estimates of the gradient, respectively; $\beta_{1}=0.9$ and $\beta_{2}=0.999$ are the exponential decay rates; ${\hat{m}}_{t}$ and ${\hat{v}}_{t}$ are the bias-corrected moment estimates; η=0.001 is the learning rate; $\epsilon =10^{-8}$ is a small constant to prevent division by zero; $\theta_{t}$ denotes the model parameters; and ∇L is the gradient of the loss function.

(3) Calculation of Information Entropy

The concept of entropy was originally derived from theoretical physics. In 1948, C. E. Shannon quantitatively described the degree of uncertainty using the probability distribution of variables and proposed the information entropy theory [27]. A higher entropy value indicates greater uncertainty in the variation in the variable, or equivalently, a larger amount of information represented. According to the type of variables, the expression of information entropy can be divided into discrete and continuous forms. Information entropy is used to quantify the uncertainty of rainfall information at a single station. The larger the information entropy, the stronger the ability of the station to capture the spatiotemporal variation characteristics of rainfall and the better its representativeness. Before calculating information entropy, the continuous rainfall series were discretized using Scott’s optimal bin width method to adaptively determine the number of discrete intervals. Based on the discretized rainfall series, the calculation formula for the information entropy of station i is as follows:

(7) $$H\left(X_{i}\right)=-\sum_{k=1}^{n}p_{ik}{log}_{2}p_{ik}$$

where $X_{i}$ denotes the (discretized) rainfall time series at station i; $p_{ik}$ is the probability that the rainfall value at station i falls into the k-th discrete interval; and n is the number of discrete intervals. During calculation, if $p_{ik}=0$, $p_{ik}{log}_{2}p_{ik}=0$ is defined as 0 to avoid the singularity of the logarithm function. Meanwhile, the information entropy of all stations is standardized (normalized to the interval [0, 1]) to facilitate subsequent coupling with other indicators. Mutual information entropy is used to quantify the overlap degree of rainfall information between two stations, serving as a core indicator for evaluating information redundancy among stations. Based on the rainfall series discretized by the discretization method, the calculation formula for the mutual information entropy $MI\left(X_{i};X_{j}\right)$ between station i and station j is as follows:

(8) $$MI\left(X_{i};X_{j}\right)=H\left(X_{i}\right)+H\left(X_{j}\right)-H(X_{i},X_{j})$$

where $H\left(X_{i}\right)$ and $H\left(X_{j}\right)$ are the information entropies of station i and station j, respectively; $H\left(X_{i},X_{j}\right)$ is the joint information entropy of station i and station j, which reflects the joint uncertainty of rainfall series at the two stations, and is calculated as:

(9) $$H\left(X_{i};X_{j}\right)=-\sum_{k=1}^{n}\sum_{l=1}^{n}p_{ikl}{log}_{2}p_{ikl}$$

where $p_{ikl}$ is the joint probability that the rainfall value of station i falls into the k-th interval and that of station j falls into the l-th interval. The mutual information entropy $I\left(X_{i};X_{j}\right)$ ranges in $\left[0,\min(H\left(X_{i}\right),H\left(X_{j}\right)\right)$. A larger value indicates a higher overlap degree of rainfall information and stronger information redundancy between the two stations. A smaller mutual information entropy implies stronger complementarity of rainfall information between the two stations, both of which should be retained to improve the information-carrying capacity of the station network.

(4) Construction of Coupled Information Entropy Index and Optimization Solution
  (a) Construction of Comprehensive Index

To achieve the core objectives of “streamlined efficiency, accurate monitoring, and low redundancy” in station network optimization, a three-dimensional optimization objective function is established, which includes prediction accuracy, information redundancy, and the number of stations. Based on this function, a fitness function is constructed as the core basis for algorithm optimization:

(10) $$\min\;F=w_{1}\cdot MSE+w_{2}\cdot (1-MI)+w_{3}\cdot \frac{N}{N_{\mathrm{total}}}$$

where F is the value of the three-dimensional optimization objective function. A smaller value indicates better overall performance of the station network, i.e., smaller prediction error, stronger spatial representativeness, and lower station cost. $w_{1},w_{2}\;and\;w_{3}$ are the weight coefficients of the three objectives. The weights were assigned according to the optimization priorities of the rain gauge network rather than being treated as purely equal coefficients. Since the primary purpose of network optimization is to maintain reliable rainfall monitoring performance after station reduction, the prediction error term was assigned the highest weight, with $w_{1}=0.4$. The information redundancy term and the station quantity term were assigned equal weights, with $w_{2}=0.3\;and\;w_{3}=0.3$, respectively, because they represent two complementary requirements: reducing redundant rainfall information and controlling the construction and maintenance cost of the network. Therefore, the adopted weight combination reflects a trade-off among accuracy preservation, redundancy reduction, and station streamlining. MSE is the prediction error of the optimized station network, representing the prediction accuracy by measuring the ability to capture spatiotemporal features. In addition, before model training and network optimization, the remote sensing precipitation product was evaluated against in situ rain gauge observations, yielding a correlation coefficient (R) of 0.82 and an RMSE of 2.31 mm/d. Therefore, the interpolation bias associated with the remote sensing product was preliminarily assessed through data validation, while the MSE term in the objective function further constrained the discrepancy between model-estimated rainfall features and observed station data. MI is the mean mutual information among all stations in the optimized network, reflecting the degree of spatial information redundancy. A smaller MI means less information overlap, stronger spatial complementarity, and better spatial representativeness among stations. N is the number of selected stations after optimization, reflecting the cost of station quantity; a smaller N corresponds to lower construction and operation costs. $N_{\mathrm{total}}$ is the original total number of stations, which is 50 in this study. N/N_“total” reflects the station reduction degree; a smaller value indicates more significant streamlining effect and better cost control.

  (b) Solution by GA-PSO Hybrid Optimization Algorithm

The GA-PSO hybrid optimization algorithm adopts a two-layer collaborative structure of “GA global search + PSO local optimization”. Its core logic is to first realize efficient global search through the selection, crossover, and mutation operations of the Genetic Algorithm [28] (GA), explore potential optimal station network schemes, and effectively prevent the algorithm from falling into local optima. Then, combined with the velocity-position update mechanism of the Particle Swarm Optimization (PSO) algorithm [29], local refined optimization is performed on the optimal solution output by GA to further improve the accuracy of station network optimization. The collaboration of the two algorithms enhances optimization efficiency and scheme rationality, ensuring that the optimized network simultaneously meets the three objectives of high prediction accuracy, low information redundancy, and reasonable number of stations [30].

Taking the fitness function as the evaluation criterion for particles (station network schemes), the fitness function is constructed combined with the three-dimensional optimization objective function, converting the multi-objective minimization problem into a fitness maximization problem. The specific formula is as follows:

(11) $$Fit=1-F=1-(w_{1}\cdot MSE+w_{2}\cdot (1-MI)+w_{3}\cdot \frac{N}{N_{\mathrm{total}}})$$

The core advantage of the fitness function lies in the organic integration of the three-dimensional optimization objectives: prediction accuracy, information redundancy, and the number of stations. It not only avoids the imbalance of station network performance caused by single-objective optimization (such as degraded prediction accuracy due to excessive streamlining of stations, or station redundancy resulting from excessive pursuit of accuracy), but also reflects the priority of each objective through weight assignment, which conforms to the optimization requirements of “streamlined efficiency and accurate monitoring” for the rain gauge network in basin. It serves as the optimization evaluation criterion for the GA-PSO hybrid optimization algorithm.

## 3. Process and Results of Station Network Layout

Based on the above methodological framework, combined with in situ data from 50 rain gauges and PERSIANN-CCS remote sensing data in the upper reaches of the Tuojiang River, the optimal layout of the rain gauge network is implemented.

### 3.1. Data Matching and Unification

First, temporal alignment is performed between the daily observed rainfall data from 50 ground rain gauges during 2015–2024 and the daily PERSIANN-CCS remote sensing rainfall data for the same period. Using ArcGIS, the remote sensing grid data are interpolated to the spatial locations of each observed station via Kriging interpolation to construct a paired “observed-remote sensing” dataset, ensuring consistency in spatiotemporal scales.

Second, multidimensional feature calculation is conducted for each station. The time-series features (mean, variance, extreme value, coefficient of variation), spatial location features (longitude, latitude, elevation, slope and aspect), and remote sensing grid features (grid rainfall mean, grid rainfall coefficient of variation) are calculated one by one, which are integrated to form a multidimensional input feature vector for each station.

Finally, dataset partitioning and standardization were carried out. Following the principle of spatiotemporal consistency, the dataset was divided into training, validation, and test sets at a ratio of 7:2:1, with continuous temporal segments used to maintain the seasonal pattern of rainfall and the continuity of rainstorm processes. During sequence generation, samples crossing the temporal splitting boundaries were excluded, and a temporal buffer with a length equal to the model’s look-back window was retained before and after each partition point to prevent data leakage among the training, validation, and test subsets. All input features were normalized to the interval [0, 1] to eliminate dimensional differences.

### 3.2. CNN-LSTM Spatial Feature Extraction

1. Model Construction. In accordance with the CNN-LSTM architecture described in Section 2.3, the preprocessed multidimensional feature vectors are used as model inputs. Spatial-related features (elevation, slope, aspect, remote sensing grid features, etc.) are fed into the CNN spatial feature extraction module, while temporal-related features (daily rainfall series, temporal statistical characteristics, etc.) are imported into the LSTM time-series modeling module. This ensures that the two modules extract features independently and lays a foundation for subsequent spatiotemporal feature fusion.
2. Model Training. The CNN-LSTM model was trained according to the strategy described in Section 2.3. In this section, the final hyperparameter configuration and training results are reported. The key hyperparameters were optimized using the grid search method, including the look-back window length, the number of CNN convolutional kernels, the number of LSTM hidden-layer units, the learning rate, and the batch size. The look-back window was tuned by considering both the physical lag-time of rainfall events in the upper Tuojiang River Basin and the validation performance of the model. Candidate window lengths were tested to balance the representation of short-term rainfall persistence and the avoidance of excessive temporal redundancy. The spatial filters in the CNN module were tuned to capture local spatial gradients and neighborhood-scale rainfall heterogeneity associated with terrain variation, including elevation, slope, aspect, and remote sensing precipitation descriptors. The final optimal hyperparameter combination was determined as follows: the numbers of kernels in the three CNN convolutional layers were 32, 64, and 128, respectively, and the LSTM hidden-layer units were 128 and 64.

**Figure 3:**
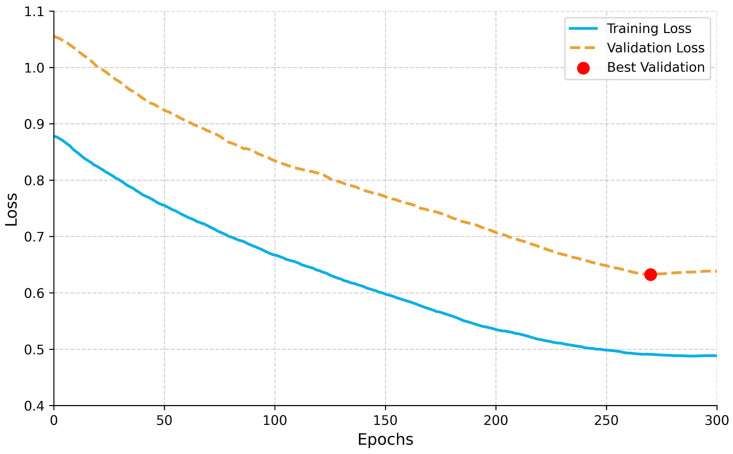
Training and validation loss curves of the spatiotemporal deep learning model.

The training process of the CNN-LSTM spatiotemporal deep model constructed in this study is shown in Figure 3. To further examine model convergence, the maximum number of training epochs was increased from 200 to 300. The training loss gradually decreased from approximately 0.88 to 0.49, while the validation loss decreased from approximately 1.06 to 0.63. After approximately 270 epochs, the validation loss gradually approached a plateau and showed no further obvious improvement, indicating that the model had reached a stable convergence state. Therefore, the model corresponding to the minimum validation loss was selected as the final model to balance fitting capacity and generalization performance.

3. Calculation of Prediction Error (MSE). After model training and validation, the preprocessed feature vectors of all 50 rain gauges are input into the trained CNN-LSTM model. Through the collaborative operation of each module, spatial and temporal features are extracted and fused. The prediction output layer then generates predicted rainfall features and observed rainfall features for each station. These values are substituted into the MSE formula to calculate the prediction error for individual stations and different station network combinations. The results serve as core input indicators for the subsequent GA-PSO hybrid optimization algorithm, providing quantitative support for the “accurate monitoring” objective of station network optimization.

### 3.3. Calculation of Information Entropy

In this study, the optimal bin width method proposed by Scott [31] is adopted to discretize the continuous rainfall time series. The core advantage of this method lies in its ability to adaptively calculate the optimal number of discrete intervals (bin value) based on the inherent distribution characteristics of rainfall data (standard deviation std(x) and total number of samples N). Compared with traditional equal-width and equal-frequency grouping methods, it can effectively avoid information loss or redundancy [32], and preserve the original spatiotemporal variability of rainfall data to the greatest extent. Moreover, the calculation process is quantifiable and reproducible, meeting the rigor requirements of academic research. Meanwhile, the optimal bin value determined by this method balances information integrity and computational efficiency, laying a reliable foundation for the subsequent unified calculation of information entropy and mutual information entropy. It is particularly suitable for the rainfall data in this study, which feature significant spatiotemporal variability and uneven distribution. The formula for the optimal number of intervals (bin value) using Scott’s method is as follows:

$${bin}_{scott}=3.49\times std\left(x\right)\times N^{-1/3}$$

where $std\left(x\right)$ is the standard deviation of the rainfall observation series x at the station, and N is the total number of samples in the rainfall data series. Based on the discretized rainfall series, the probability pik that the rainfall value of each station falls into each discrete interval is counted and substituted into the information entropy formula to calculate the information entropy H(Xi) of each station, followed by normalization to the interval [0, 1].

The joint probability $p_{ikl}$ that rainfall values of any two stations fall into corresponding discrete intervals is counted to compute the joint information entropy $H\left(X_{i};X_{j}\right)$. The mutual information entropy $MI\left(X_{i};X_{j}\right)$ between the two stations is then obtained using the mutual information formula. Finally, the mean mutual information $\bar{MI}$ among all stations is derived.

**Figure 4:**
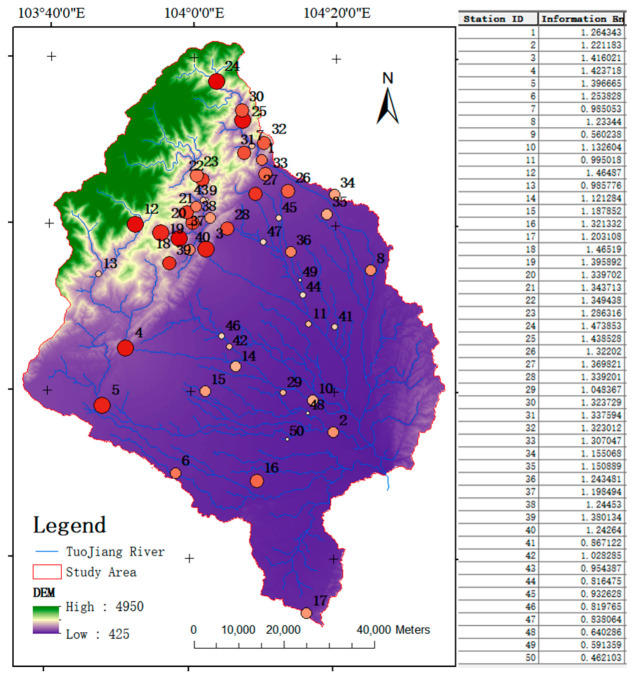
Information entropy of rain gauge stations in the study area.

**Figure 5:**
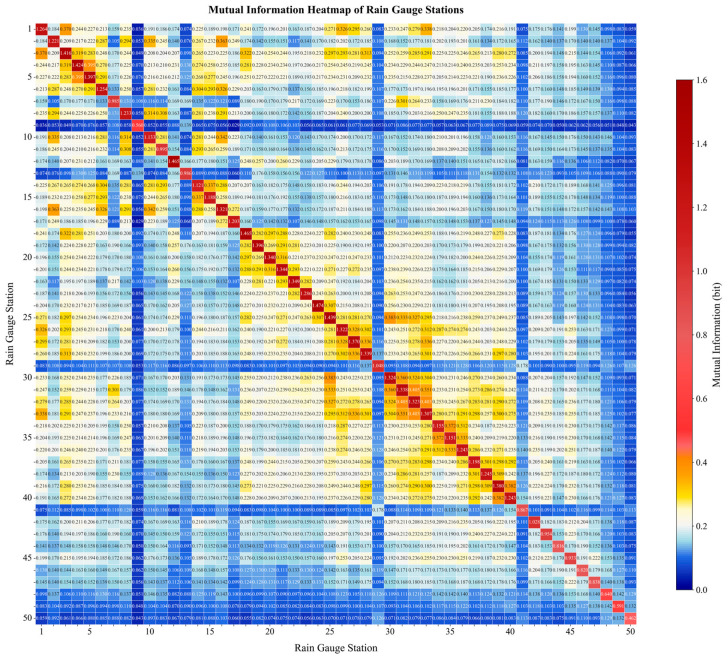
Heat map of mutual information entropy among rain gauge stations.

The calculated information entropy of each monitoring station and the mutual information between stations are shown in Figure 4 and Figure 5. The information entropy of individual stations ranges from 0.462 to 1.474 bits, with a mean value of 1.164 bits. Spatially, stations in high-altitude areas generally exhibit higher information entropy, while those in low-altitude areas are relatively lower. High-altitude regions are characterized by large topographic relief and stronger spatiotemporal variability of rainfall, leading to richer information content. In contrast, low-altitude areas have relatively stable rainfall processes and higher information redundancy. The mutual information between stations ranges from 0.029 to 0.405 bits, with a mean value of 0.177 bits. Stations with close spatial locations and similar elevation conditions tend to show stronger information correlation and rainfall synchronism, reflecting obvious information-sharing characteristics within the station network. In the mutual information heatmap, the diagonal represents the information entropy of each station itself, and the off-diagonal regions intuitively show the difference in the strength of information correlation between stations, which can clearly reveal the spatial information structure and redundancy distribution of all rain gauge stations in the study area.

### 3.4. Solution by GA-PSO Hybrid Optimization Algorithm

1. Parameter Initialization. Core parameters of GA and PSO are initialized as follows: GA parameters: population size = 50, maximum iteration = 50, crossover probability = 0.7, mutation probability = 0.4 (bit-flipping mutation). PSO parameters: number of particles = 20, maximum iteration = 10, inertia weight w = 0.5, acceleration coefficients c1 = 0.8 and c2 = 0.9.
2. Fitness Calculation. Combined with the previously calculated prediction error (MSE), mean mutual information between stations (MI), and number of stations (N), the fitness value of each individual (station network scheme) is computed using the fitness function.
3. GA Global Search. GA iteration is initiated. Through selection (randomly sampling 3 individuals each time and selecting the one with optimal fitness for the next generation), crossover (parental gene recombination), and mutation operations, the optimal station network scheme in the GA stage is output after 50 iterations.
4. PSO Local Optimization. The optimal solution from GA is taken as the initial position of the first particle, and 20 binary-coded particles are initialized. The velocity update formula of PSO is adopted as follows:

(12) $$\begin{array}{c} v_{ij}\left(t+1\right)=w\cdot v_{ij}\left(t\right)+c1\cdot r1\cdot \left(pbest_{ij}-x_{ij}\left(t\right)\right)+c2\cdot r2\\ \cdot \left(gbest_{j}-x_{ij}\left(t\right)\right) \end{array}$$

and the position update formula: $x_{ij}\left(t+1\right)=x_{ij}\left(t\right)+v_{ij}\left(t+1\right)$.

Iteration is performed for 10 rounds, with the individual optimum and global optimum updated in real time.

**Figure 6:**
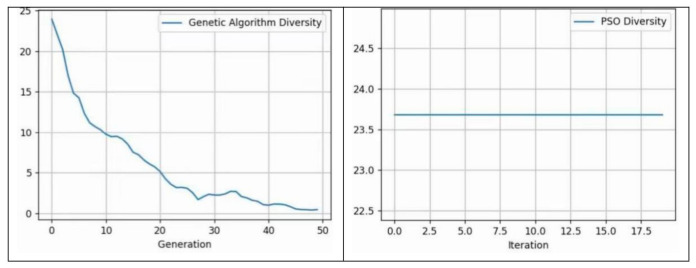
Population diversity curve of the GA-PSO algorithm.

The population diversity variation in the GA-PSO hybrid optimization algorithm is displayed in Figure 6. The population diversity of the Genetic Algorithm (GA) declines rapidly and then converges slowly with the increase in iterations. The initial diversity is about 24, which drops rapidly to below 5 within 20 generations and fluctuates slightly at a low level thereafter, reflecting the GA characteristic of “global exploration in the early stage and local convergence in the later stage”, which can efficiently accomplish global optimization of station network schemes. The population diversity of the Particle Swarm Optimization (PSO) remains at a very high level between 23.6 and 23.7 during iteration with almost no attenuation, indicating that PSO maintains strong global exploration ability in the local optimization stage and can effectively prevent the algorithm from falling into local optima. The complementary diversity features of the two algorithms verify the rationality of the GA-PSO hybrid design of “global search + local refined optimization”.

**Figure 7:**
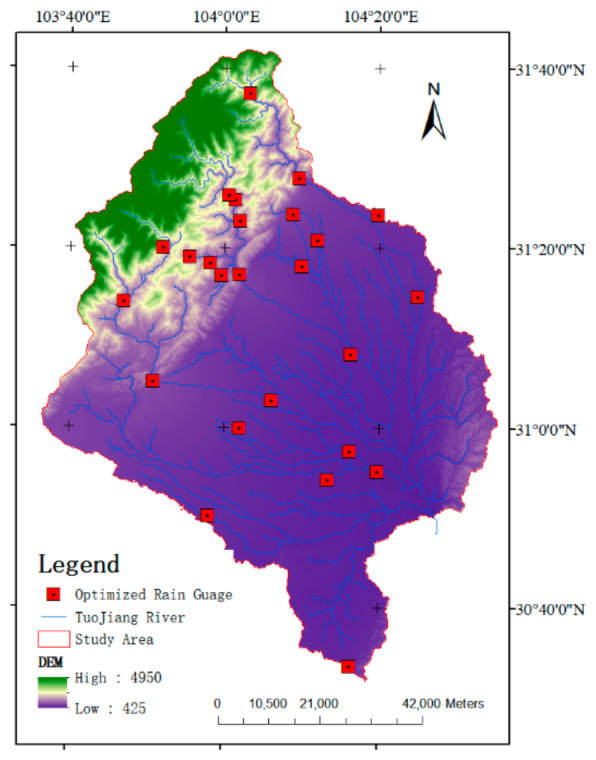
Layout results of the rain gauge network in this study.

5. Output of Optimized Rain Gauge Network Layout. After PSO iteration is completed, the global optimal solution is output as the final layout scheme for rain gauge network optimization in the upper Tuojiang River, achieving the coordinated goals of streamlined network structure, low redundancy, and high monitoring accuracy. The optimized results of the rain gauge network in the study area are shown in Figure 7.

## 4. Comparison and Discussion

To verify the effectiveness of the hybrid optimization algorithm driven by the spatiotemporal deep model coupled with mutual information (hereinafter referred to as the proposed method) in rain gauge network layout optimization, the classic greedy optimization algorithm based on mutual information entropy (hereinafter referred to as the traditional method) in the field of rain gauge network optimization is selected as the comparison benchmark. The two methods are tested in the same study area, with the same dataset and under identical constraints (i.e., consistent number of stations, identical spatial extent, and unified rainfall time series). A systematic comparison of the optimization results is conducted from four key aspects: spatial network structure, spatial rainfall distribution, mean relative error (MRE), and total information entropy of the station network.

### 4.1. Comparison of Spatial Network Structure

**Figure 8:**
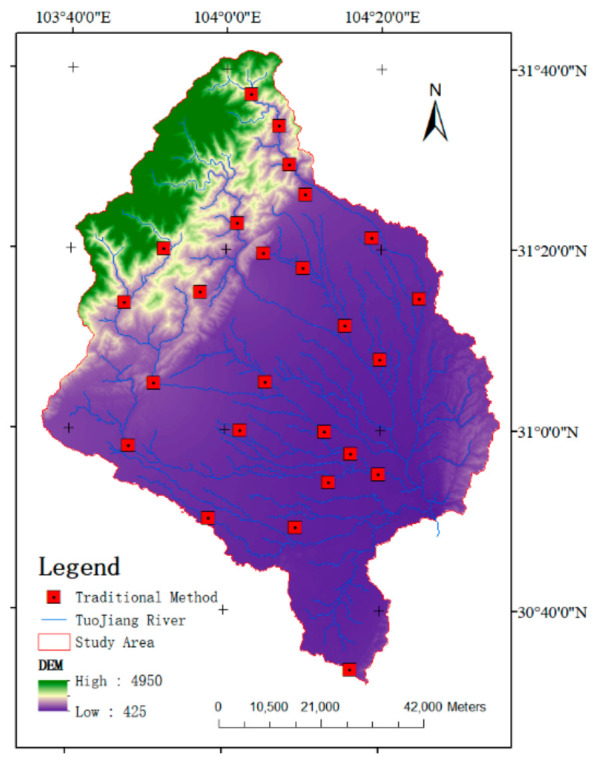
Layout results of the greedy optimization algorithm based on mutual information entropy.

The rationality of the spatial station network structure directly affects the regional rainfall coverage capability and monitoring uniformity, which serves as a fundamental indicator for evaluating the optimization effect of rain gauge network layout. By comparing the two optimized layouts in Figure 7 and Figure 8, geographically, stations optimized by both methods achieve effective coverage of the upper Tuojiang River study area with generally uniform distribution and no obvious over-concentration or blank regions. The traditional method yields a uniformly distributed layout with satisfactory geographic coverage. Stations optimized by the proposed method are also evenly distributed overall; meanwhile, according to actual characteristics of the study area, relatively dense station clusters are formed in the northern zone with large elevation variations. In terms of river network correlation, stations from the traditional method are mostly located near the main stream and tributaries, with some sited at river confluences, effectively capturing ambient rainfall responses around water bodies. Stations from the proposed method also follow river trends, with the core advantage being denser distribution at key hydrological nodes such as river confluences, providing more targeted monitoring for core catchment areas and enabling more accurate capture of spatiotemporal rainfall variations therein. Regarding reasonability of station density, the traditional method maintains uniform density across the entire region with only moderate intensification at important geographic nodes like river confluences. In contrast, the proposed method presents distinct regional differentiation: denser in the northwest and relatively sparse in the southeast. Moreover, it allocates more high-density stations at critical geographic nodes such as river confluences than the traditional method, supporting more precise monitoring of regional rainfall amount and spatiotemporal dynamics to obtain more representative observation data. In comparison, although the traditional method achieves full regional coverage, it suffers from slight under-coverage in parts of the northern study area and insufficient station density at key monitoring zones such as river confluences, leading to limited refinement in monitoring. The proposed method enhances targeting and completeness of coverage by strategically densifying stations in critical areas, resulting in significantly higher refinement than the traditional method. In summary, the proposed method demonstrates superior performance in distribution targeting, density rationality, and coverage refinement. It better adapts to the geographic features, hydrological conditions, and practical monitoring demands of the study area, showing a more reasonable layout effect.

### 4.2. Comparison of Spatial Rainfall Distribution

**Figure 9:**
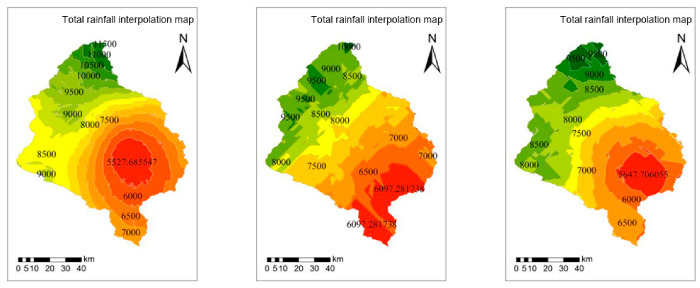

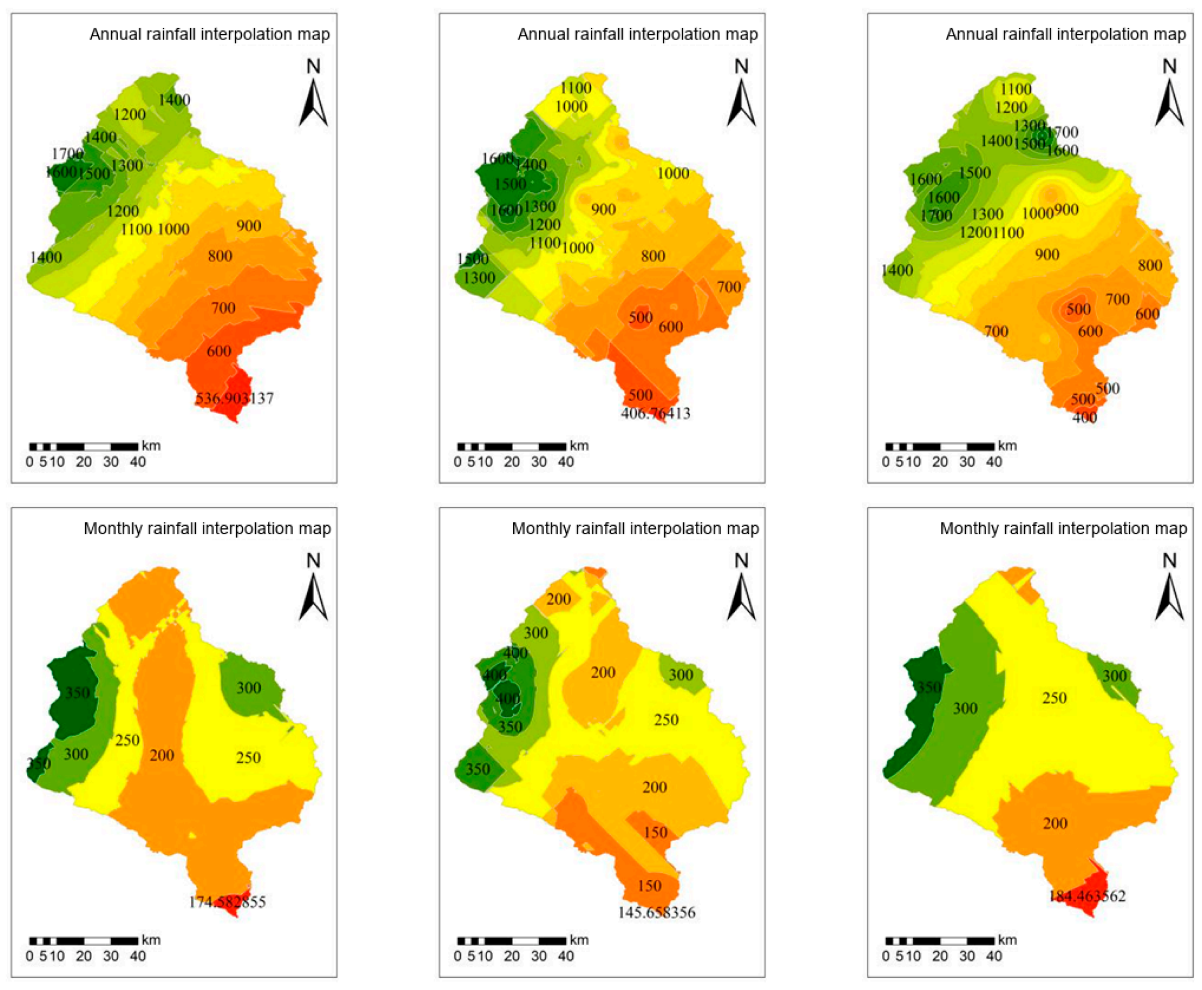
Kriging interpolation maps of rainfall before and after station network optimization. ((**left column**) Rainfall interpolation results of all 50 rain gauges; (**middle column**) Rainfall distribution captured by the proposed station network; (**right column**) Rainfall distribution captured by the traditional station network).

The characterization accuracy of spatial rainfall distribution is a core objective for evaluating the optimization effect of rain gauge network layout, whose accuracy directly depends on the network’s ability to capture spatiotemporal rainfall characteristics. Based on the station network data optimized by the two methods, the Kriging interpolation method is used to generate spatial rainfall distribution maps of the study area. The total rainfall, annual rainfall, and monthly rainfall corresponding to the optimized networks are calculated separately (Figure 9). By comparing the interpolation results and map characteristics, the spatial rainfall depiction performance of the two methods is systematically analyzed.

From the comparison of rainfall interpolation maps at different time scales, the two methods show significant differences in interpolation performance, and the proposed method is generally superior. On the total rainfall and annual rainfall interpolation maps, the results of the proposed method can accurately present the spatial gradient characteristics of rainfall, with contour distributions relatively consistent with the actual regional rainfall patterns. The total rainfall map of the traditional method suffers from insufficiently smooth rainfall transitions. Although its annual rainfall map shows relatively natural transitions, it fails to fully capture the spatial heterogeneity of regional rainfall. The main reason is that the traditional method does not fully consider spatiotemporal rainfall correlation and only pursues local information gain, making the interpolation results difficult to match the actual regional rainfall characteristics. On the monthly rainfall interpolation maps, the proposed method performs prominently in rainfall continuity and isosurface regional distribution, clearly reflecting spatial differences in monthly rainfall. This benefits from the coupling of mutual information and the spatiotemporal deep model in the proposed method, which accurately captures spatiotemporal rainfall features. The optimized network layout better conforms to regional rainfall distribution laws, thereby improving interpolation performance. In contrast, limited by the greedy strategy, the traditional method does not fully consider spatiotemporal rainfall correlation and inter-station synergy, resulting in unsatisfactory interpolation effects. In summary, the proposed method achieves higher accuracy in depicting spatial rainfall characteristics, which further verifies its effectiveness.

### 4.3. Comparison of Mean Relative Error

The mean relative error (MRE) is a core indicator for quantifying the observation accuracy of a station network. Its magnitude directly reflects the deviation between the observed data of the optimized network and the original rainfall data. A smaller MRE value indicates higher consistency between the rainfall data captured by the optimized network and the original data, implying better optimization performance. Conversely, a larger MRE value suggests more significant deviation, representing unsatisfactory optimization results.

To objectively evaluate the optimization effects of the proposed method and the traditional method, this study takes the upper reaches of the Tuojiang River as the research area and conducts statistical analysis at three time scales: total rainfall from 2015 to 2024, annual average rainfall from 2015 to 2024, and monthly average rainfall. The areal average rainfall and mean relative error of the networks optimized by the two methods are calculated, with the detailed statistical results shown in Table 1.

According to the table data, the proposed method shows better overall interpolation accuracy than the traditional method at the total-period and monthly average rainfall scales. In terms of total rainfall from 2015 to 2024, the total rainfall of the original station network is 8524.52 mm. After optimization by the proposed method, it is 8163.73 mm, with an MRE of 4.24%. By contrast, the traditional method yields 7662.44 mm, with an MRE of 10.12%. Therefore, the MRE of the proposed method is reduced by 57.9% compared with the traditional method. At the monthly average rainfall scale, the MRE values are consistent with those of total rainfall: the proposed method achieves an MRE of 4.24%, whereas the traditional method produces an MRE of 10.12%.

At the annual rainfall scale from 2015 to 2024, the proposed method achieved lower MRE values than the traditional method in four years, namely 2016, 2017, 2018, and 2019. In particular, the optimization effect in 2018 was the most prominent, with an MRE of only 0.62%, much lower than the 21.31% obtained by the traditional method. However, relatively large deviations were observed in 2015 and 2022, indicating that the performance of the optimized network may still be affected by interannual differences in rainfall magnitude and spatial distribution. Therefore, in addition to the total-period and monthly scale MRE comparison, a leave-one-year-out robustness validation was further conducted to examine whether the proposed method was overfitted to specific climatic cycles within the 10-year dataset.

In the leave-one-year-out validation, each year from 2015 to 2024 was sequentially excluded from model training and station-network optimization and was used only as an independent test year. The traditional mutual-information-entropy-based greedy optimization method was also evaluated under the same setting for comparison. The results are shown in Table 2.

The leave-one-year-out validation results show that the proposed method achieved a mean MRE of 11.03%, which was lower than that of the traditional mutual-information-entropy-based greedy optimization method (13.86%). This corresponds to a relative reduction of approximately 20.4%, indicating that the proposed framework maintained reasonable cross-year robustness under interannual rainfall variability. Although the MRE of the proposed method was relatively high in 2015, the original annual evaluation in Table 1 also showed a large deviation for this year, suggesting that 2015 may represent a difficult year for precipitation reconstruction due to its specific rainfall magnitude and spatial distribution characteristics. Excluding 2015, the mean MRE of the proposed method decreased to 8.39%, further supporting its stable performance in most years.

### 4.4. Comparison of Station Network Information Entropy

The total information entropy of a station network serves as a key metric for evaluating its information acquisition capability. A higher total entropy value indicates a stronger capacity to capture regional rainfall dynamics and more comprehensive information coverage, thereby reflecting a more scientifically sound and rational network layout. To rigorously compare the differences in information acquisition capacity between the proposed method and the traditional approach, the total information entropy of the networks optimized by both methods was calculated based on monitoring data from the upper Tuojiang River study area. The detailed statistical results are presented in Table 3.

As indicated by the data in Table 3, a comparison of metrics such as station count, information entropy, and redundancy reveals that both the proposed and traditional optimization methods successfully streamline the original 50 stations to 25. Although they perform equally well in achieving the targeted station reduction, significant differences emerge regarding information utilization efficiency, the extent of redundancy reduction, and overall representativeness.

(1) The total information entropy of the station network characterizes the overall capacity of the network to capture rainfall information. A higher entropy value indicates a greater total information volume and a stronger ability to depict the spatiotemporal variability of rainfall within the basin. The original 50-station network yields a total information entropy of 95.1706 bits. After optimization via the proposed method, the 25-station network achieves a total information entropy of 46.9153 bits, outperforming the 44.7836 bits obtained by the traditional method. Retaining a higher total information volume while halving the number of stations demonstrates that the stations selected by the proposed method contribute more comprehensively and exhibit stronger representativeness of the basin’s rainfall patterns.
(2) The joint information entropy reflects the combined uncertainty and overall informational richness collectively provided by all stations, demonstrating the network’s informational completeness as an integrated system. The proposed method yields an optimized joint information entropy of 9.7978 bits, which is higher than the 9.5939 bits achieved by the traditional method. This suggests that the network optimized by the proposed method possesses a more robust overall information structure and enhanced synergistic observation capabilities among station combinations, allowing for a more comprehensive delineation of the spatial distribution patterns of rainfall.
(3) The average mutual information entropy measures the degree of rainfall information overlap among stations. A larger value denotes higher information redundancy, whereas a smaller value implies stronger informational complementarity. The network optimized by the proposed method exhibits an average mutual information entropy of 0.2264 bits, slightly higher than the 0.2164 bits from the traditional method. This indicates that while the proposed method effectively reduces redundancy, it refrains from over-minimizing mutual information. Instead, it strikes a balance between total information volume and spatial representativeness, resulting in a more uniform station layout and a more scientifically sound observation structure.
(4) Redundant information entropy, defined as the difference between total and joint information entropy, represents the volume of duplicated and overlapping invalid information within the network. A lower redundancy entropy signifies a more streamlined network and a more efficient layout. The redundant information entropy of the original network reaches as high as 84.4625 bits, which drastically decreases to 37.1175 bits after optimization using the proposed method, significantly mitigating information overlap. Compared to the traditional method, the magnitude of redundancy reduction in the proposed method is slightly smaller. This is because the proposed approach prioritizes the retention of high-information stations over the mere minimization of redundancy, thereby maximizing the preservation of valid information while streamlining the stations.

In summary, the hybrid optimization approach combining CNN-LSTM and GA-PSO proposed in this study successfully reduces the number of stations from 50 to 25 while achieving a higher total information entropy, a more complete joint information structure, and a reasonable reduction in redundancy. Compared to the traditional method, it achieves a superior balance among “station streamlining, information preservation, and redundancy control.” Consequently, the optimized network exhibits higher information utilization efficiency and stronger spatial representativeness, making it highly applicable to the practical demands of regional rainfall monitoring.

Synthesizing the comparative analysis from the preceding four subsections, the hybrid optimization algorithm driven by a spatiotemporal deep model coupled with mutual information, as proposed in this study, demonstrates significant superiority in optimizing rain gauge network layouts. Its core advantages stem from two aspects. First, the integration of the spatiotemporal deep model with mutual information accurately captures the spatiotemporal correlations within rainfall data, quantifies information interaction and redundancy among stations, and thus provides a more robust scientific and theoretical foundation for network optimization. Second, the hybrid optimization strategy overcomes the local optimum limitations inherent in traditional greedy algorithms, achieving a globally optimal layout that effectively balances spatial uniformity, observation accuracy, and information acquisition capability.

The limitations of traditional methods primarily arise from their greedy optimization strategies, which solely prioritize the information gain of individual stations while neglecting the spatiotemporal characteristics of rainfall and the synergistic effects among stations. Consequently, this leads to an imbalanced spatial layout characterized by the coexistence of information redundancy and monitoring blind spots, ultimately compromising the network’s observation accuracy and information acquisition capacity. By employing a multi-dimensional optimization design, the proposed method effectively resolves these issues, achieving a scientifically sound and rational rain gauge network layout. Furthermore, the optimization results highlight that an optimal rain gauge network is not merely a uniform distribution of stations. Instead, it necessitates the integration of regional spatiotemporal rainfall heterogeneity and topographical features to fulfill the objective of “precise layout and efficient monitoring.”

It should also be noted that the use of remote sensing precipitation products may introduce interpolation bias between satellite-based estimates and ground-based observations. In the current optimization framework, this bias was evaluated through comparison with in situ rain gauge observations and was indirectly constrained by the prediction-error term, but it was not explicitly incorporated as an independent objective function. This may affect the accuracy of the spatiotemporal rainfall features extracted by the CNN-LSTM model and the subsequent station evaluation. Future work will consider introducing a dedicated interpolation-bias term or constructing a multi-objective function that explicitly minimizes interpolation bias together with prediction error, information redundancy, and station cost.

## 5. Conclusions

As a critical support for hydrological and water resource monitoring, flood control, disaster mitigation, and water resource regulation, the rational layout of rain gauge networks serves as the core prerequisite for ensuring the accurate acquisition of precipitation data and enhancing monitoring cost-effectiveness. To this end, this study integrates the information quantification advantages of mutual information entropy with the feature-fitting capabilities of the CNN-LSTM spatiotemporal deep model to construct a hybrid optimization algorithm coupled with mutual information entropy. Taking the upper reaches of the Tuojiang River as the study area, and utilizing in situ rain gauge data and PERSIANN-CCS remote sensing precipitation products from 2015 to 2024, a comparative experiment against the traditional mutual information entropy-based greedy optimization algorithm was conducted to systematically verify the feasibility and superiority of the proposed method.

The research indicates that the constructed CNN-LSTM spatiotemporal deep model effectively parses the spatial distribution disparities and temporal evolution patterns of rainfall data, providing reliable spatiotemporal feature support for network layout optimization. Building upon this, the hybrid optimization algorithm, coupled with mutual information entropy metrics, achieves the scientific optimization of the rain gauge network layout. Following the application of this optimization algorithm, the original 50 rain gauges in the upper Tuojiang River can be streamlined to 25. This significantly reduces costs associated with network deployment, operation, and maintenance, while effectively preserving the core characteristics of the basin’s spatiotemporal rainfall distribution.

Compared to the traditional mutual information entropy-based greedy algorithm, the proposed optimization method exhibits distinct advantages across multiple dimensions. The optimized spatial structure of the network better conforms to the geographical and hydrological characteristics of the upper Tuojiang River basin, yielding a more precise representation of spatial rainfall distribution. The mean relative error at the monthly average precipitation scale is 4.24%, and the total information entropy of the network is increased compared to pre-optimization levels, reflecting a significant enhancement in information acquisition capability. These findings fully demonstrate that the proposed CNN-LSTM hybrid optimization algorithm coupled with mutual information entropy not only provides a practical technical solution for the optimal layout of the rain gauge network in the upper Tuojiang River, but also offers a theoretical reference for similar basin network optimization efforts. This holds profound practical significance for improving basin water resource monitoring efficiency and refining water resource management systems.

The core reason the proposed optimization method outperforms traditional algorithms lies in the synergistic enhancement between the CNN-LSTM model and mutual information entropy. The CNN-LSTM model deeply mines the intrinsic spatiotemporal correlations of rainfall data, accurately capturing rainfall distribution patterns. Meanwhile, mutual information entropy effectively quantifies the complexity of rainfall information and the information redundancy among stations. Combined with a hybrid optimization strategy, this approach achieves a globally optimal network layout, effectively resolving the core pain point of traditional greedy algorithms—the inability to simultaneously balance station streamlining and monitoring accuracy. Even after the number of stations is reduced to 25, the monitoring accuracy continues to satisfy the practical requirements of basin hydrological monitoring, a result that fully corroborates the economic viability and practical utility of the proposed method.

Nevertheless, this study is subject to certain limitations. The research scope is currently confined to the upper reaches of the Tuojiang River basin. Future work could extend this method to basins with diverse climate types and topographical conditions, and incorporate specific hydrological scenarios such as extreme rainfall and floods, to further validate the method’s universality and adaptability. Additionally, incorporating underlying surface factors of the basin, such as topographical slope and soil moisture content, could further improve the model’s feature-fitting precision. Moreover, economic indicators such as network deployment costs and operational maintenance efficiency could be integrated to construct a multi-objective optimization framework, thereby enhancing the method’s engineering applicability. In conclusion, the optimization algorithm proposed in this study effectively addresses the deficiencies of traditional rain gauge network optimization methods and provides a novel technical pathway for the optimal layout of basin rain gauge networks. By further refining the model structure and expanding its application scenarios in the future, this method is poised to play a crucial role in water resource monitoring and management across broader basins, offering robust technical support for flood control, disaster mitigation, and the rational planning and regulation of water resources.

## Figures and Tables

**Table 1:** Comparison of rainfall interpolation accuracy between two optimization methods.

Temporal Scale	Kriging Interpolation Result/mm			Mean Relative Error/%	
	Original Rainfall Station	Proposed Method	Traditional Method	Proposed Method	Traditional Method
2015–2024 Total precipitation	8524.52	8163.73	7662.44	4.24	10.12
Mean annual precipitation in 2015	348.43	460.22	314.03	32.08	9.87
Mean annual precipitation in 2016	619.07	644.03	530.86	4.03	14.25
Mean annual precipitation in 2017	653.00	700.93	539.12	7.34	17.44
Mean annual precipitation in 2018	1049.28	1055.81	825.70	0.62	21.31
Mean annual precipitation in 2019	647.71	597.80	572.75	7.71	11.57
Mean annual precipitation in 2020	1170.10	1073.94	1103.74	8.22	5.67
Mean annual precipitation in 2021	1064.42	956.50	983.02	10.14	7.65
Mean annual precipitation in 2022	949.83	822.18	844.40	13.44	11.10
Mean annual precipitation in 2023	888.65	821.54	845.06	7.55	4.91
Mean annual precipitation in 2024	1134.01	1030.78	1103.74	9.10	2.67
Mean monthly precipitation	71.04	68.03	63.85	4.24	10.12

**Table 2:** Leave-one-year-out validation results of the proposed and traditional methods.

Leave-Out Year	MRE of Proposed Method (%)	MRE of Traditional Method (%)
2015	34.81	14.23
2016	6.12	16.72
2017	7.48	20.88
2018	1.31	25.61
2019	8.02	14.32
2020	9.23	8.53
2021	11.14	11.61
2022	14.36	13.76
2023	7.61	7.38
2024	10.24	5.54
Mean	11.03	13.86

**Table 3:** Statistics of total information entropy of station networks.

	All Stations in the Study Area	Optimized Station Network in This Study	Station Network Optimized by Traditional Methods
Number of stations	50	25	25
Total information entropy of the station network (bit)	95.1706	46.9153	44.7836
Joint information entropy of the station network (bit)	10.7081	9.7978	9.5939
Average mutual information entropy of the station network (bit)	0.2462	0.2264	0.2164
Redundant information entropy of the station network (bit)	84.4625	37.1175	35.1897

## Data Availability

The raw data supporting the conclusions of this study are not publicly available due to relevant data confidentiality restrictions, but can be obtained from the corresponding author upon reasonable request.

## References

[1] Mishra A.K., Coulibaly P. (2009). Developments in hydrometric network design: A review. Rev. Geophys..

[2] Wang J., Chen J., Shen P., Guan X., Liu X., Massari C., Wang Z., Feng M., Wang Q., Lu Y. (2025). Regional-scale intelligent optimization and topography impact in restoring global precipitation data gaps. Commun. Earth Environ..

[3] Suri A., Azad S. (2024). Optimal placement of rain gauge networks in complex terrains for monitoring extreme rainfall events: A review. Theor. Appl. Climatol..

[4] Li D., Wang J., Deng K., Zhang D., Zhao C., Leng H., Wen Y., Liu Y., Ren K., Song J. (2026). Review on deep learning quantitative precipitation nowcasting: Advances and challenges. Expert Syst. Appl..

[5] Feki H., Slimani M., Cudennec C. (2016). Geostatistically based optimization of a rainfall monitoring network extension: Case of the climatically heterogeneous Tunisia. Hydrol. Res..

[6] Wang K., Chen N., Tong D., Wang K., Wang W., Gong J. (2015). Optimizing precipitation station location: A case study of the Jinsha River Basin. Int. J. Geogr. Inf. Sci..

[7] Li C., Singh V.P., Mishra A.K. (2012). Entropy theory-based criterion for hydrometric network evaluation and design: Maximum information minimum redundancy. Water Resour. Res..

[8] Alfonso L., He L., Price R., Lobbrecht A. (2013). Information theory applied to evaluate the discharge monitoring network of the Magdalena River. J. Hydroinformatics.

[9] Huang Y., Zhao H., Jiang Y., Lu X. (2020). A method for the optimized design of a rain gauge network combined with satellite remote sensing data. Remote Sens..

[10] Chin W.F.L., Suhaili W.S. (2023). Rain gauge network optimization in Brunei-Muara district using historical data. Proceedings of the 6th International Conference on Applied Computational Intelligence in Information Systems (ACIIS), Bandar Seri Begawan, Brunei, 23–25 October 2023.

[11] Haggag M., Elsayed A.A., Awadallah A.G. (2016). Evaluation of rain gauge network in arid regions using geostatistical approach: Case study in Northern Oman. Arab. J. Geosci..

[12] Skok G. (2006). Analytical and practical examples of estimating the average nearest-neighbor distance in a rain gauge network. Meteorol. Z..

[13] Shi X., Chen Z., Wang H., Yeung D.Y., Wong W.K., Woo W.C. (2015). Convolutional LSTM network: A machine learning approach for precipitation nowcasting. Adv. Neural Inf. Process. Syst..

[14] Shi X., Gao Z., Lausen L., Wang H., Yeung D.Y., Wong W.K., Woo W.C. (2017). Deep learning for precipitation nowcasting: A benchmark and a new model. Adv. Neural Inf. Process. Syst..

[15] Chen S., Xu X., Zhang Y., Shao D., Zhang S., Zeng M. (2022). Two-stream convolutional LSTM for precipitation nowcasting. Neural Comput. Appl..

[16] Zhang T., Liang Z., Bi C., Wang J., Hu Y., Li B. (2025). Statistical post-processing for precipitation forecast through deep learning coupling large-scale and local-scale spatiotemporal information. Water Resour. Manag..

[17] Xu C., Liu J., Han S., Duan X., Xiang L., Zhang T. (2025). FourCastLSTM: A precipitation nowcasting model integrating global and local spatiotemporal features. Comput. Geosci..

[18] Nourani V., Tosan M., Huang J.J., Gebremichael M., Kantoush S.A., Dastourani M. (2025). Advances in multi-source data fusion for precipitation estimation: Remote sensing and machine learning perspectives. Earth Sci. Rev..

[19] Yeh H.C., Chen Y.C., Chang C.H., Ho C.H., Wei C. (2017). Rainfall network optimization using radar and entropy. Entropy.

[20] Dai Q., Bray M., Zhuo L., Islam T., Han D. (2017). A scheme for rain gauge network design based on remotely sensed rainfall measurements. J. Hydrometeorol..

[21] Hsu K.L., Gao X., Sorooshian S., Gupta H.V. (1997). Precipitation estimation from remotely sensed information using artificial neural networks. J. Appl. Meteorol..

[22] Zeweldi D.A., Gebremichael M. (2009). Sub-daily scale validation of satellite-based high-resolution rainfall products. Atmos. Res..

[23] Cánovas-García F., García-Galiano S., Karbalaee N. (2017). Validation of a global satellite rainfall product for real time monitoring of meteorological extremes. Proceedings of the SPIE 10421, Remote Sensing for Agriculture, Ecosystems, and Hydrology XIX, Warsaw, Poland, 11–14 September 2017.

[24] Teegavarapu R.S.V., Chandramouli V. (2005). Improved weighting methods, deterministic and stochastic data-driven models for estimation of missing precipitation records. J. Hydrol..

[25] Moritz S., Bartz-Beielstein T. (2017). imputeTS: Time series missing value imputation in R. R J..

[26] Arellano C.J., Dao V., Gorooh V.A., Alharbi R.S., Nguyen P. (2023). Performance of the PERSIANN family of products over the Mekong River Basin and their application for the analysis of trends in extreme precipitation indices. Atmosphere.

[27] Shannon C.E. (1948). A mathematical theory of communication. Bell Syst. Tech. J..

[28] Holland J.H. (1975). Adaptation in Natural and Artificial Systems: An Introductory Analysis with Applications to Biology, Control, and Artificial Intelligence.

[29] Kennedy J., Eberhart R. (1995). Particle swarm optimization. Proceedings of the IEEE International Conference on Neural Networks, Perth, WA, Australia, 27 November–1 December 1995.

[30] Settles M., Soule T. (2005). Breeding swarms: A GA/PSO hybrid. Proceedings of the 7th Annual Conference on Genetic and Evolutionary Computation (GECCO ’05), Washington, DC, USA, 25–29 June 2005.

[31] Scott D.W. (1979). On optimal and data-based histograms. Biometrika.

[32] Huang Y., Zhao H., Jiang Y., Lu X., Hao Z., Duan H. (2019). Comparison and analysis of different discrete methods and entropy-based methods in rain gauge network design. Water.

